# Supplementary material for: A vignette study of option refusal and decision deferral as two forms of decision avoidance: Situational and personal predictors
Source: PLoS One. 2020 Oct 23;15(10):e0241182. doi: 10.1371/journal.pone.0241182 (PMC7584223; doi:10.1371/journal.pone.0241182)
Supplement: S1 File — (DOCX) [file pone.0241182.s002.docx]

**Overview of variables in the dataset**

**Sociodemographics:**

- Age: 18-40
- Gender: 1=female, 2=male
- Education: 1=A level, 2= university degree bachelor, 3=university degree master, 4=PhD degree
- Psychology student: 0=no, 1=yes

**Other data_long format**: long data format for mixed-method models

- Code: Participants (each participant got eight vignettes of one decision situation: seminar_at_university, plans_for_evening, internship, student_apartment; 0=no, 1=yes)
- Randomization: 1= seminar_at_university, 2=plans_for_evening, 3=internship, 4=student_apartment

**Distribution decision avoidance** (non-hierarchical data format): Within persons (=codes), how many decisions have been deferred, refused or have been made?

- decision_deferral_number (values 0-8)
- option_refusal_number (values 0-8)
- decision_made_number (values 0-8)
- decision_not_made_number (values 0-8)

**Variables in the ‘Other data_long format’ file:**

Control variables:

- Seriousness (value 0-101): how seriously the study was handled by the participants?
- process_time_overall (values in seconds): how fast participants filled in the survey?
- previous_experiences (value 0-101): Did participants have previous experiences with similar decision making situations in the past?
- reference_to_reality (value 0-101): How realistically were the decision making situations experienced?

Main dependent variables

- Deferralvsdecision: 1=decision deferral, 0=decision made, 99=option refusal
- option_refusalvsdecision: : 1= option refusal, 0=decision made, 99=decision deferral

Further dependent variables:

- Difficulty (value between 0-101): difficulty of decision
- Satisfaction(value between 0-101): satisfaction with decision or avoidance behavior (subjective indicator for decision quality)

Independent variables (manipulated situational factors):

- attractiveness: 0=low attractiveness of the choice-set, 1=high attractiveness of the choice set
- lack_of_information: 0=low lack of information, 1=high lack of information
- time_pressure: 0=low time pressure, 1=high time pressure

Further independent variables for multi-level-models:

- Zimportance: importance of the decision (z-transformed value), (previously value 0-101)
- Zsimilarity: similarity of the alternatives (z-transformed value), (previously value 0-101)
- Zdifficulty: difficulty of decision (z-transformed value), (previously value 0-101)
- Zprocrastination_indecisiveness: decision style chronic indecisiveness (z-transformed value), (previously value 5-15)

Decision styles (personality variables)

- Confident: Decision style
- Vigilant: Decision style
- Intuitive: Decision style
- Dependent: Decision style
- Buckpassing: Decision style
- Procrastination_indecisiveness: Decision style
- Anxious: Decision style
- NCC: need for cognitive closure
